# Effect of denosumab on the incidence of fractures and mortality in patients undergoing hemodialysis: A retrospective cohort study

**DOI:** 10.1371/journal.pone.0309657

**Published:** 2024-08-29

**Authors:** Arisa Kobayashi, Tatsuhiro Yaginuma, Kazuhiko Kato, Akio Nakashima, Ichiro Ohkido, Takashi Yokoo

**Affiliations:** 1 Division of Nephrology and Hypertension, Department of Internal Medicine, Jikei University School of Medicine, Tokyo, Japan; 2 Human Health Sciences, Kyoto University Graduate School of Medicine, Kyoto, Japan; 3 Division of Nephrology, Keijin Hospital, Tokyo, Japan; Mayo Clinic Rochester, UNITED STATES OF AMERICA

## Abstract

**Background:**

Patients undergoing hemodialysis are at an elevated risk of fractures; however, substantial evidence for osteoporosis treatment in this population is lacking. We explored the efficacy of denosumab, an anti-IgG2 antibody that targets the receptor activator of nuclear factor-kappa B ligand, in reducing fracture incidence and all-cause mortality in patients undergoing hemodialysis.

**Methods:**

This retrospective cohort study—conducted from December 2013 to December 2022—evaluated the effects of denosumab on fracture incidence and all-cause mortality. Patients who initiated denosumab treatment during the study period were defined as the denosumab group, while those without a history of denosumab administration were defined as the non-denosumab group. Kaplan–Meier curves and log-rank tests were used to assess survival and fracture/mortality risks, respectively. Cox proportional hazards models were used to analyze both fractures and all-cause mortality.

**Results:**

Among 214 patients undergoing hemodialysis, 52 (24.3%) received denosumab. The median age was 73.0 ± 11.5 years, with 92 (43.0%) females, and the median dialysis duration was 59 months (interquartile range, 6–126). During the study, thirty-seven non-denosumab-treated patients had fractures compared to eight in the denosumab group. No significant differences were observed in the unadjusted model (HR, 0.53; 95% confidence interval (CI), 0.24–1.14). Adjusting for competing mortality and clinical factors, the HR remained at 0.64 (95% CI, 0.27–1.51). Regarding all-cause mortality, we found a statistically significant difference in the unadjusted model (HR, 0.61 [95% CI, 0.38–0.98]). A significant reduction in mortality was observed in the adjusted model (HR, 0.46 [95% CI, 0.26–0.80]). Notably, the denosumab group showed a significant decrease in mortality, particularly in cardiovascular disease-related cases (HR, 0.33 [95% CI, 0.14–0.78]).

**Conclusions:**

Denosumab may reduce all-cause mortality in patients undergoing hemodialysis, particularly in those with cardiovascular complications. This finding offers a promising direction for osteoporosis treatment in patients undergoing hemodialysis.

## Introduction

Patients undergoing hemodialysis are at an increased risk of bone fractures, with reported incidences approximately 4–7 times higher than those in the general population [1–3]. Furthermore, patients undergoing hemodialysis who develop fractures are at a higher risk of subsequent death. Fractures not only decrease patients’ quality of life but can also lead to increased complications and death. Preventing the onset of fractures and taking appropriate countermeasures are important [4]. Many osteoporosis medications are contraindicated or require careful administration in patients undergoing dialysis [5]. Consequently, these patients may not receive sufficient treatment for osteoporosis.

Denosumab, a potent human monoclonal anti-IgG2 antibody, targets receptor activators of the nuclear factor-kappa B ligand (RANKL) with remarkable precision, effectively thwarting its interaction with human RANKL through high-affinity binding. In recent years, denosumab has gained widespread usage and has exhibited strong inhibition of bone resorption in the general population, underscoring its efficacy [6]. This strategic interference effectively suppresses osteoclast formation, imparting a robust inhibitory effect on bone resorption [7].

Several studies have investigated the effects of denosumab on surrogate markers like bone mineral density (BMD) and bone turnover markers in patients undergoing hemodialysis. However, to date, no study has explored the association between denosumab use and bone fractures in this patient population [8–12]. Moreover, existing evidence indicates a positive correlation between pre-treatment bone turnover markers and the response to denosumab treatment [13]. However, these studies included few participants and had short observational periods. Furthermore, no study has investigated the relationship between bone fractures and denosumab use in patients undergoing hemodialysis. Therefore, this retrospective cohort study aimed to investigate the effects of denosumab on bone fractures and the prognosis of patients undergoing hemodialysis.

## Methods

### Study design

This retrospective cohort study was approved by the Institutional Review Board of Jikei University (Approval No. 34-387(11544)). Our research is an observational study focused on hemodialysis patients; therefore, individual consent was not required for this study, as approved by the IRB at our facility. Opt-out opportunities were provided to each participant and documented on the website of the responsible research institution, allowing individuals the option to decline participation. This study complied with the principles of the Declaration of Helsinki.

The primary outcome was the occurrence and cause of death and the secondary outcome was the occurrence of new fractures in the entire body, including the femur, thoracolumbar spine, pelvis, and other sites.

### Data source and patient selection

This cohort study included inpatients and outpatients aged ≥ 20 years undergoing hemodialysis at Keijin Hospital, a maintenance hemodialysis facility in Tokyo, Japan. The study period extended from December 2013 to December 2022. Patients undergoing dialysis at Keijin Hospital during this period and who were diagnosed with osteoporosis were included in this study. The diagnosis of osteoporosis followed the Japanese guidelines established by the Japanese Osteoporosis Society [5]. In essence, patients were diagnosed with osteoporosis if their BMD was less than 70% of young adult mean (YAM) or if their BMD was less than 80% with a history of fragility fractures. Patients younger than 20 years of age and those using drugs for osteoporosis other than denosumab were excluded from this study. We anonymized the personally identifiable information of the study participants during data collection, and did not access personally identifiable information during or after data collection. Patients who initiated denosumab treatment during the study period were categorized into the denosumab group, whereas those without denosumab administration comprised the non-denosumab group. Denosumab was administrated based on meeting diagnostic criteria for osteoporosis, absence of contraindications, patient consent, and physician approval. The treatment regimen consisted of a 60 mg subcutaneous injection administered every six months.

### Clinical parameters

Data were collected from routine dialysis laboratory records. Blood samples obtained at the start of each week were included as part of routine clinical examinations for all patients. Parameters including albumin (g/dL), urea nitrogen (mg/dL), creatinine (mg/dL), calcium (mg/dL), phosphate (mg/dL), magnesium (mg/dL), and intact parathyroid hormone (PTH) (pg/mL) levels were assessed using standard commercial assays. Routine laboratory parameters were evaluated using standard commercial assays. The number of missing values for clinical variables included in the models were as follows: TRACP5b had 9 missing values, BAP had 8 missing values, and all other blood test data were complete.

BMD was assessed using dual-energy X-ray absorptiometry (DEXA) with a HITACHI Dichroma Scan, DCS-600 EXV. Baseline BMD was measured at the distal third of the radius before treatment in the treated group and at study entry in the untreated group. BMD measurements were also systematically performed in all patients. We also reviewed current medications, such as active vitamin D analogs, calcium carbonate, phosphate binders, calcimimetics, and angiotensin-converting enzyme (ACE) inhibitors or angiotensin II receptor blockers. Data regarding deaths and causes of death were collected from medical records. The decision to use denosumab was made by the attending physician, considering the guidelines for osteoporosis, and evaluating each individual case.

### Statistical analysis

Demographic data are presented as mean ± standard deviation or median (interquartile range [IQR]) for continuous variables and numerical values (percentages) for categorical variables. Differences in background characteristics between the two groups were assessed using the chi-square test for binary variables and the t-test for continuous variables. We utilized Kaplan–Meier curves to calculate survival probabilities and performed log-rank tests to evaluate disparities in fracture and mortality risks between the denosumab-treated and denosumab-naive groups. To address potential confounders, we conducted further adjustments for age, sex, history of bone fractures, PTH levels, and BMD levels as explanatory variables. Subsequently, we employed a competing risk regression analysis using the Fine and Gray model to adjust for the competing risk of death when assessing the risk of fractures. For survival analysis, Cox regression was employed to examine all deaths using age, sex, diabetes mellitus (DM), dialysis vintage, history of bone fractures, and levels of serum albumin, PTH, Ca, phosphate, C-reactive protein, and active vitamin D analogs as explanatory variables. Furthermore, we generated Kaplan–Meier curves and performed Cox regression analysis to examine the specific causes of death, such as cardiovascular disease (CVD) and infectious diseases. This analysis used age, sex, DM, and serum albumin level as explanatory variables to investigate the associations with each cause of death. We conducted a sensitivity analysis employing propensity score matching to strengthen our findings. The level of statistical significance was set at p<0.05 for all tests. Statistical analyses were performed using STATA version 17.0 (StataCorp LLC, College Station, TX, USA).

## Results

### Patient background

We excluded two eligible patients with missing age and sex information, resulting in a final analysis of 214 patients. Fifty-two patients (24.3%) were treated with denosumab, and 162 patients (75.7%) were not treated with denosumab. Table 1 presents the patient characteristics and clinical parameters. The median age was 73.0 ± 11.5 years, and 92 patients (43.0%) were female. The median history of dialysis was 59 months (IQR, 6–126 months). A total of 116 patients (54.5%) had DM. A history of fractures was observed in 47 patients (29.0%) in the non-denosumab group and 23 patients (15.4%) in the denosumab group. Serum phosphorus and intact PTH levels did not differ between the groups and were controlled within the appropriate ranges.

**Table 1:** Characteristics by group.

	Total population	non-denosumab	denosumab	P Value
	N = 214	N = 162	N = 52	
Age [years]	73.0 ± 11.5	72.7 ± 12.2	74.0 ± 9.0	0.50
Female [n (%)]	92 (43.0)	60 (37.0)	32 (61.5)	0.002
Dialysis vintage [months]	59 (6–126)	54 (4–122)	62 (24–142)	0.16
Diabetes mellitus [n (%)]	116 (54.5)	95 (59.0)	21 (40.4)	0.019
Cancer [n (%)]	18 (8.4)	14 (8.6)	4 (7.7)	0.83
PTX [n (%)]	5 (2.3)	3 (1.9)	2 (3.8)	0.41
Previous fractures [n (%)]	70 (32.7)	47 (29.0)	23 (44.2)	0.042
Incidence fractures [n (%)]	45 (21.0)	37 (22.8)	8 (15.4)	0.25
Bone Mineral Density [g/cm^2^]	0.52 (0.41–0.63)	0.57 (0.42–0.65)	0.46 (0.39–0.52)	<0.001
Albumin [g/dL]	3.4 (2.9–3.8)	3.3 (2.8–3.7)	3.6 (3.1–4.0)	0.008
BUN [mg/dL]	52.7 (40.0–65.8)	51.5 (38.4–65.6)	57.8 (47.9–65.9)	0.041
Cr [mg/dL]	7.28 (5.28–9.16)	7.02 (5.22–9.01)	7.97 (5.75–9.66)	0.11
Ca [mg/dL]	8.4 (8.0–8.8)	8.4 (7.9–8.7)	8.5 (8.2–9.0)	0.022
P [mg/dL]	4.5 (3.6–6.2)	4.3 (3.8–5.3)	4.55 (3.5–5.2)	0.94
Mg [mg/dL]	2.4 (2.1–2.6)	2.3 (2.1–2.6)	2.5 (2.3–2.7)	0.007
iPTH [pg/mL]	104 (62–190)	113 (65–187)	86 (48–197)	0.46
TRACP-5b [mU/dL]	523 (341–734)	504 (337–712)	595 (400–778)	0.18
BAP [μg/L]	15.35 (12.2–21.1)	14.6 (11.9–19.9)	18.1 (15.1–25.1)	<0.001
Active vitamin D analogue [n (%)]	132 (61.7)	85 (52.5)	47 (90.4)	<0.001
CaCO3 [n (%)]	75 (35.0)	43 (26.5)	32 (61.5)	<0.001
Non-calcium-containing phosphate binder [n (%)]	52 (24.3)	37 (22.8)	15 (28.8)	0.38
Calcimimetics [n (%)]	86 (40.2)	57 (35.2)	29 (55.8)	0.008
ACE/ARB [n (%)]	123 (57.5)	86 (53.1)	37 (71.2)	0.022

Continuous variables are summarized as medians (interquartile ranges), and categorical variables are presented as numbers of patients (%). Bone mineral density was measured at the distal 1/3 of the radius. Abbreviations: PTX, parathyroidectomy; BUN, blood urea nitrogen; Cr, creatinine; Ca, calcium; P, phosphate; Mg, magnesium; iPTH, intact parathyroid hormone; TRACP–5b, Tartrate—resistant acid phosphatase 5b; BAP, Bone specific alkaline phosphatase; CaCO3, Calcium carbonate; ACE, angiotensin—converting enzyme (ACE) inhibitor; ARB, angiotensin II receptor blocker.

Patients treated with denosumab utilized a higher quantity of vitamin D preparations than untreated patients. This trend stems from the fact that these preparations are frequently initiated at the onset of treatment to mitigate denosumab-induced hypocalcemia.

### Safety and adverse events

Following denosumab administration, serum calcium levels decreased to their lowest point, with a median of 7.8 mg/dL (95% confidence interval (CI), 7.2–8.6) one week later, and returned to baseline levels. Most cases were asymptomatic, except for one case of tetany, which resolved with calcium supplements and active vitamin D analogue. No atypical fractures were reported in this study, and none of the patients in the denosumab group required treatment interruption.

### Risk of developing fractures

Throughout the study period, fractures occurred in 37 patients (22.8%) in the non-denosumab treatment group, whereas only 8 patients (15.4%) experienced fractures in the denosumab group. We examined fractures within a maximum of 9 years after the initial denosumab administration. The median time to fracture occurrence after denosumab administration was 27.5 months (95% CI, 8.0–45.5) in the denosumab group. Regarding fracture sites, 13 patients (28.9%) experienced femoral fractures, 14 (31.1%) suffered vertebral fractures, 3 (6.7%) encountered pelvic fractures, and 19 (42.2%) incurred fractures in other sites such as radial and humeral fractures. Four patients experienced fractures at two different sites.

**Fig 1:**
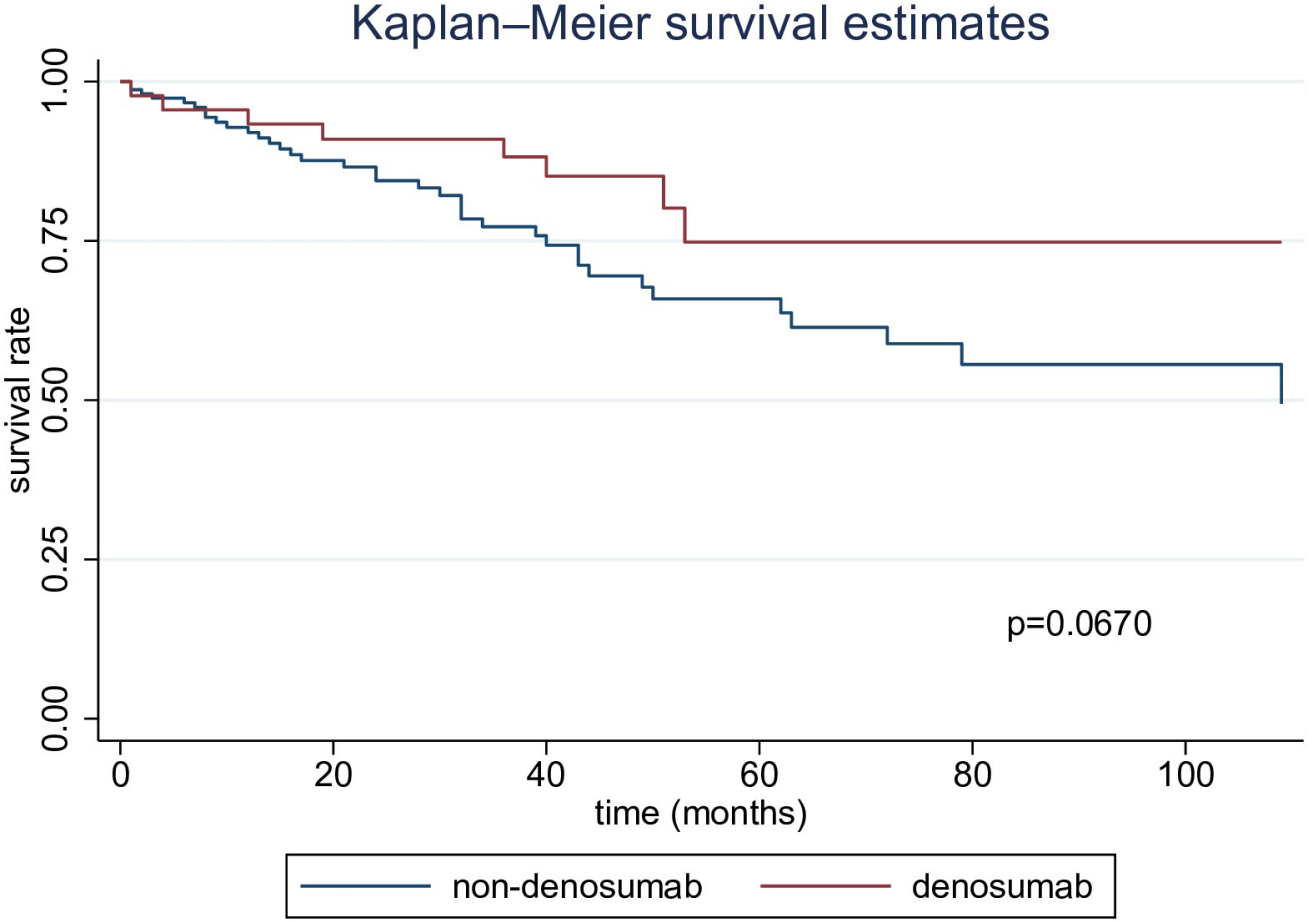
Kaplan–Meier curves showing the onset of fractures.

Examining the impact of denosumab administration on fracture occurrence, the log-rank test indicated that the incidence of fractures was not significantly lower in the denosumab group compared to the non-denosumab group (p = 0.0670) (Fig 1). Moreover, the competing risks regression analysis using the Fine and Gray model showed no significant difference in fracture risk in the unadjusted group (Hazard ratio (HR), 0.53 [95% CI, 0.24–1.14]). After adjusting for competing mortality as well as age, sex, history of bone fractures, BMD and PTH levels, the HR was 0.64 (95% CI, 0.27–1.51), indicating no statistically significant difference between the two groups (Table 2).

**Table 2:** Risk of fracture with competing risk in denosumab users versus non-users.

	Events [n (%)]		Hazard Ratio (95%CI)	P Value
Non-denosumab	60 (37.0)		reference	
Denosumab	21 (40.4)	Unadjusted	0.53 (0.24–1.14)	0.104
		Adjusted	0.64 (0.27–1.51)	0.310

adjusted: Age, sex, history of bone fractures, bone mineral density, intact parathyroid hormone added to the unadjusted model

Additionally, we separately analyzed fracture incidence based on sex, age, and PTH levels. We found no significant differences between males and females, patients aged below and above 75 years, or those in the high- and low-PTH groups.

### Survival analysis

**Fig 2:**
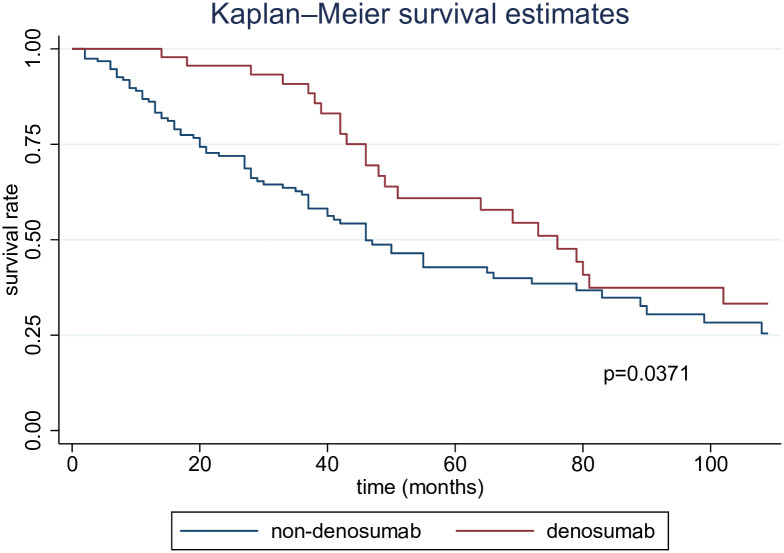
Kaplan–Meier curves for all-cause mortality.

We examined the effects of denosumab on mortality. We found a statistically significant difference in mortality between the two groups (p = 0.0371) (Fig 2). Furthermore, cox regression analysis adjusted for each explanatory variable indicated a notable reduction in mortality within the denosumab group (HR, 0.46 [95% CI, 0.26–0.80]) (Table 3).

**Table 3:** Survival analysis in denosumab users versus non-users.

		Events [n (%)]		Hazard Ratio (95%CI)	P Value
All-cause mortality	Non-denosumab	78 (48.1)		reference	
	Denosumab	23 (44.2)	Unadjusted	0.61 (0.38–0.98)	0.040
			Adjusted*	0.46 (0.26–0.80)	0.006
CVD	Non-denosumab	3 (1.9)		reference	
	Denosumab	8 (15.4)	Unadjusted	0.54 (0.25–1.17)	0.120
			Adjusted**	0.33 (0.14–0.78)	0.011
Infectious disease	Non-denosumab	26 (16.0)		reference	
	Denosumab	8 (15.4)	Unadjusted	0.63 (0.29–1.41)	0.263
			Adjusted**	0.53 (0.21–1.31)	0.169

Abbreviation: CVD, cardiovascular disease.

adjusted*: Age, sex, diabetes mellitus, dialysis vintage, history of bone fractures, albumin levels, intact parathyroid hormone, calcium, phosphate, C-reactive protein, and vitamin D medication added to the unadjusted model

adjusted**: Age, sex, diabetes mellitus and albumin levels added to the unadjusted model

**Fig 3:**
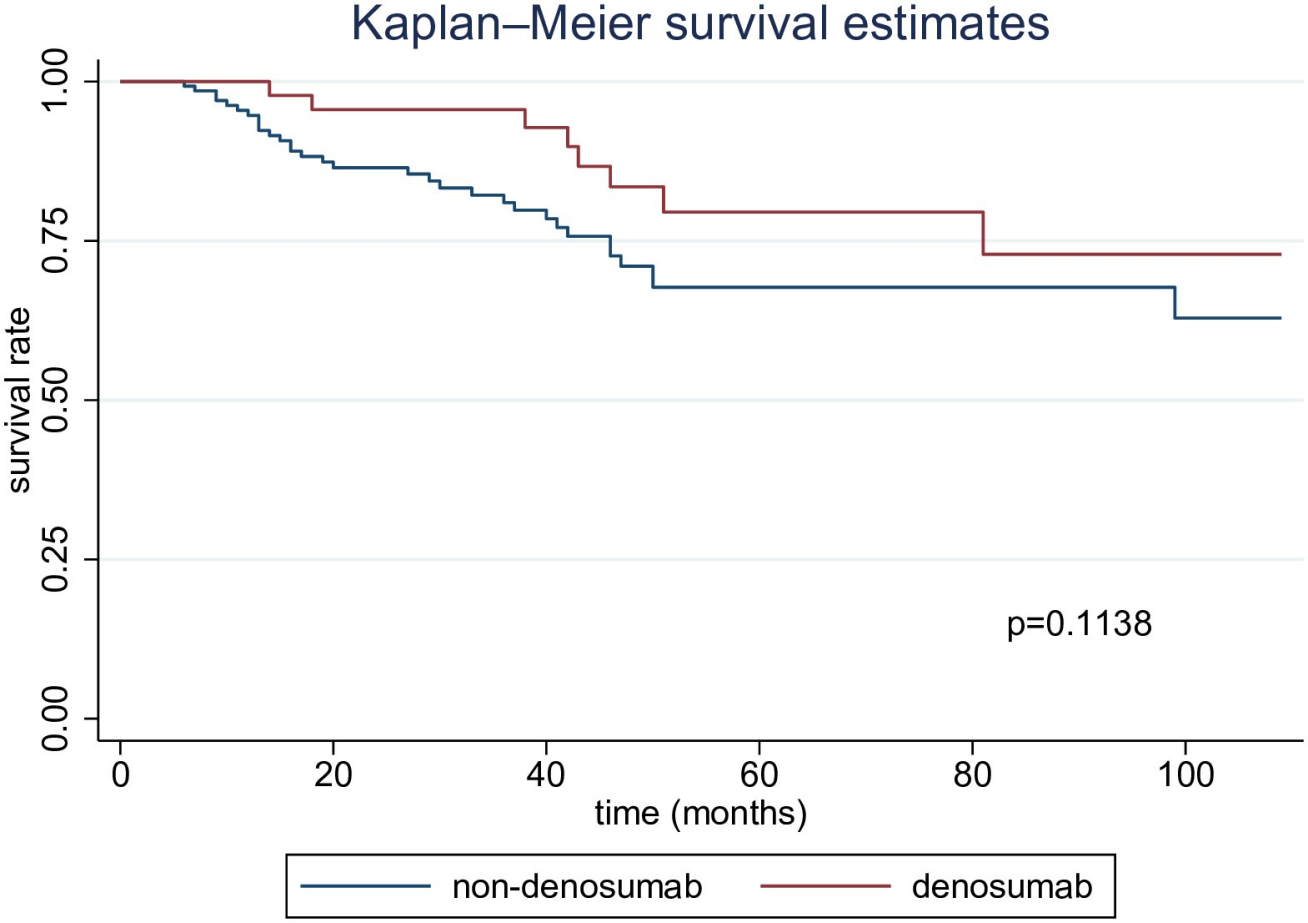
Kaplan–Meier curve for mortality related to CVD.

**Fig 4:**
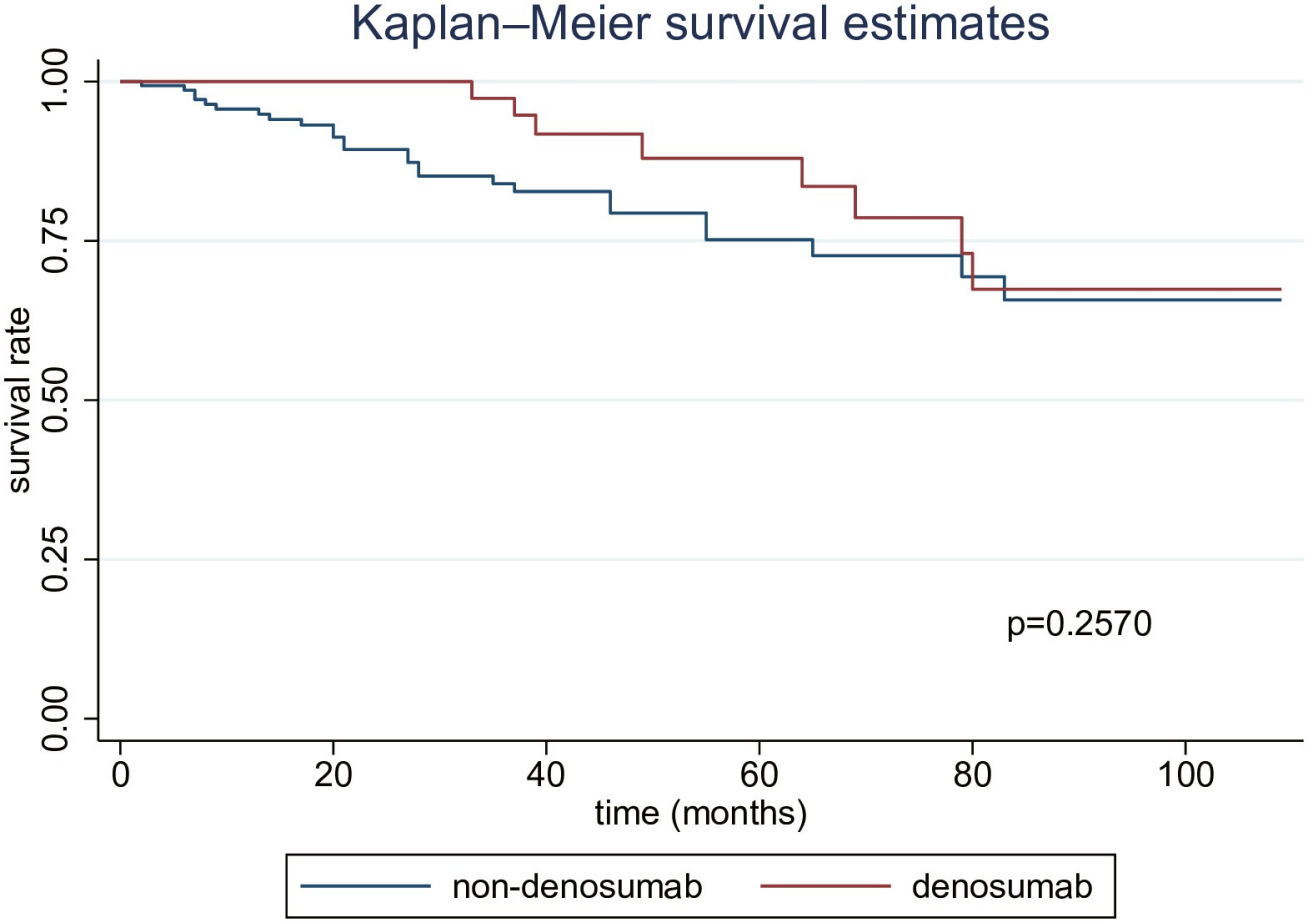
Kaplan–Meier curves for mortality related to infectious disease.

Additional analyses were conducted based on the cause of death. However, no clear or statistically significant differences were observed in mortality related to CVD or infectious diseases (Figs 3 and 4). Conversely, upon adjusting for certain explanatory variables in the Cox regression analysis, we found a significant decrease in mortality linked to CVD (HR, 0.33 [95% CI, 0.14–0.78]). In contrast, no statistically significant difference was observed in deaths attributed to infectious diseases, even following adjustment (HR, 0.55 [95% CI, 0.22–1.34]) (Table 3).

### Sensitivity analysis

We conducted a sensitivity analysis using propensity score matching to validate our findings further. This method incorporated age, sex history of bone fractures, BMD and PTH levels, to create two comparable groups: those treated with denosumab and those who did not receive denosumab. The analysis included survival curves for both fracture incidence and mortality. Additionally, we employed a competing risks regression analysis using the Fine and Gray model to address the competing risk of death when evaluating fracture risk. The log-rank test indicated that the incidence of fractures did not significantly differ between the denosumab and non-denosumab groups (p = 0.4908) (S1 Fig). Furthermore, the competing risks regression analysis using the Fine and Gray model showed no significant difference in fracture risk within the unadjusted group (HR, 1.93 [95% CI, 0.40–9.28]).

In contrast, the analysis of overall mortality revealed a notable decrease in death rates among patients receiving denosumab (S2 Fig). Cox regression analysis indicated a significant reduction in mortality within the denosumab group (HR, 0.38 [95% CI, 0.21–0.70]). Further analyses focusing on causes of death demonstrated a statistically significant difference in mortality related to CVD, whereas no significant differences were observed in mortality related to infectious diseases (S3 and S4 Figs). Similarly, Cox regression analysis highlighted a significant decrease in CVD-related mortality (HR, 0.30 [95% CI, 0.11–0.77]), while no significant difference was found in deaths attributed to infectious diseases (HR, 0.47 [95% CI, 0.15–1.46]).

## Discussion

In this study, denosumab demonstrated efficacy in reducing mortality among patients undergoing hemodialysis; however, its impact on fractures remains inconclusive. Furthermore, a significant reduction in CVD-related mortality was observed in patients receiving denosumab. To the best of our knowledge, this study represents the first to elucidate the long-term effects of denosumab on mortality in patients undergoing hemodialysis. Nevertheless, it is essential to exercise caution when interpreting findings because the Kaplan–Meier curves cross over at certain points in time. We speculate that the difference in patient backgrounds between the two groups and the potential variation in the effect of denosumab administration in the early and late stages may explain this phenomenon. This limitation is inherent in our study.

Bone loss is associated with increased all-cause and CVD mortality in patients with end-stage renal failure [14]. In addition to improving BMD and preventing bone loss, an important outcome is preventing fractures, as fracture development has been shown to increase subsequent mortality and complications in patients undergoing dialysis [15]. Therefore, the treatment of osteoporosis in patients undergoing dialysis is one of the most critical concerns.

Several studies have documented the use of denosumab in patients undergoing dialysis. Previous research demonstrating the efficacy of denosumab in this population often focused on BMD as a primary outcome measure due to the relatively short follow-up period. The pharmacokinetics of denosumab circumvents renal excretion, rendering its exposure in patients with renal failure almost on par with those with normal renal function [16]. Therefore, denosumab has emerged as a viable therapeutic option [17]. Furthermore, reports indicate that denosumab usage in patients undergoing hemodialysis enhances BMD in the hip region, affecting both cortical and trabecular bones. Continued administration of denosumab is anticipated to lead to ongoing improvements and maintenance of bone density [11]. However, our study did not observe a significant reduction in the incidence of fractures. Several factors could contribute to this outcome: (I) the limited number of cases; (II) the predominant inclusion of elderly patients in the study population, with mortality potentially occurring before the onset of fractures; (III) the inclusion of both outpatients and inpatients, who may exhibit distinct clinical characteristics; and (IV) our analysis encompassed all fractures without specific focus on fractures such as those in the femur or thoracolumbar spine. Addressing the decline in BMD in dialysis patients is complex. We hypothesized that the observed decrease in BMD in these patients may be influenced by various factors beyond secondary hyperparathyroidism (SHPT). These factors include natural aging, which naturally decreases bone density; poor nutritional status, affecting bone health; the effects of dialysis treatment; decreased physical activity levels; chronic inflammation typical in dialysis patients; and the presence of diabetes mellitus. These factors collectively contribute to bone mineral loss, indicating a multifaceted issue rather than one solely dependent on SHPT.

Our study showed that denosumab significantly reduced the mortality rate of patients undergoing hemodialysis. In particular, deaths due to CVD were suppressed. These mechanisms may be related to the RANKL/RANK/osteoprotegerin (OPG) pathway, which plays numerous roles in various organs and involves bone metabolism [18]. OPG is abundantly expressed in various tissues, including bones and blood vessels, and neutralizes RANKL, which is associated with bone metabolism and suppresses bone resorption [19]. It is a known regulator of vascular calcification and plays a protective role [19]. Denosumab binding to RANKL may mimic the action of OPG and inhibit vascular calcification [20]. Moreover, in previous clinical trials, denosumab reduced aortic calcification in patients undergoing hemodialysis [21] and cardiovascular calcification in patients with secondary hyperparathyroidism undergoing hemodialysis [22]. Denosumab also reportedly reduced the risk of death and ischemic stroke in females with osteoporosis compared to raloxifene [23]. These mechanisms suggest that the therapeutic effect of denosumab also reduces vascular calcification, leading to a significant reduction in mortality. Among osteoporosis medications, those that inhibit bone rotation may help reduce vascular calcification.

It has also been reported that fracture reduction leads to mortality reduction, suggesting that osteoporosis treatment may reduce fractures and improve the ability to recover from acute illnesses [24]. The reduction in fractures may have led to the suppression of death in addition to reducing cardiovascular mortality. However, no significant difference was observed in the fracture incidence.

We also investigated the sex-specific effects of denosumab treatment. Previous studies have suggested differences in fracture risk between sexes in patients undergoing hemodialysis, with implications of sex hormones [25, 26]. Denosumab, which is anticipated to be efficacious in postmenopausal women owing to increased RANKL signaling consequent to reduced gonadal function, may have amplified the efficacy among women within this predominantly older patient cohort. No statistically significant differences in fractures were observed in our study, warranting further exploration with an expanded case pool. However, concerning mortality, the denosumab group exhibited a significant reduction in fractures among female patients, with a notable difference observed in patients aged 75 years and older. This suggests that denosumab may be an effective treatment for osteoporosis in older females undergoing dialysis. This may be due to the involvement of sex hormones or differences in general conditions, including the effects of age. In contrast, subgroup analyses in previous studies examining the effect of denosumab treatment in postmenopausal patients with osteoporosis showed no differences according to age [27]. Further studies are required to confirm this hypothesis.

This study has several limitations. First, this was a single-center observational study, which limited the number of cases. Consequently, we were not able to analyze site-specific incidences of fractures. This is a significant limitation of our study, as many studies have examined site-specific incidences of fractures when evaluating the effects of osteoporosis drugs. We aim to accumulate more cases to address this issue. Second, the attending physician made the decision to administer denosumab, and the criteria for this decision were primarily based on their judgment. Therefore, selection bias in drug use may have occurred. Although selecting only patients diagnosed with osteoporosis may help reduce the impact of selection bias, it cannot be completely eliminated. Future studies exploring the efficacy of refined intervention guidelines should enhance therapeutic accuracy. Third, this study was conducted at a facility that included both inpatients and outpatients. In particular, inpatients are likely to exhibit different activities of daily living, clinical backgrounds, and characteristics compared to outpatients, which might have influenced the results. In future studies, we aim to augment the sample size and analyze both inpatient and outpatient cases. Fourth, bone density was measured in the distal third of the distal radius. Although the femur is generally recommended for bone density measurement, bone density measurement of the distal radius is useful in outpatient maintenance hemodialysis facilities, where it is easier to assess. Therefore, our study may be helpful in clinical and other treatment settings. In addition, this study did not include BMD, bone metabolic markers, or bone biopsy results. Improvements in BMD and bone metabolic markers may have contributed to the suppression of fracture risk; however, we did not ascertain this. Moreover, data could not be obtained during the analysis period. Specifically, the content and dosage of drug therapy, and laboratory values during the study should be regarded as potential factors influencing the development of cardiovascular disease. We were not able to procure information concerning cardiovascular risk scores and frailty, which are both mortality indicators. Furthermore, it was not possible to assess ionized calcium levels. Additional analyses of these factors will be conducted in the future. While it is necessary to consider these limitations, an important clinical endpoint is a reduction in the incidence of bone fractures and mortality. We believe that this study is valuable because it allowed long-term observation of the incidence of bone fractures and mortality from the start of denosumab treatment.

In conclusion, denosumab suppresses mortality in patients undergoing hemodialysis and may be an effective treatment option for osteoporosis. Further studies are required to confirm these results, including the effects of fracture suppression.

## Supporting information

S1 Fig Kaplan–Meier curves showing the onset of fractures between two propensity score-matched groups. (TIF)

S2 Fig Kaplan–Meier curves for all-cause mortality between two propensity score-matched groups. (TIF)

S3 Fig Kaplan–Meier curves for CVD-related mortality between two propensity score-matched groups. (TIF)

S4 Fig Kaplan–Meier curves for infectious disease-related mortality between two propensity score-matched groups. (TIF)
