# Correction: Effect of denosumab on the incidence of fractures and mortality in patients undergoing hemodialysis: A retrospective cohort study

**DOI:** 10.1371/journal.pone.0320569

**Published:** 2025-03-21

**Authors:** Arisa Kobayashi, Tatsuhiro Yaginuma, Kazuhiko Kato, Akio Nakashima, Ichiro Ohkido, Takashi Yokoo

In Table 3, the number of CVD cases in the non-denosumab group should be corrected from “3 (1.9)” to “32 (19.8)”. Please see the correct Table 3 here.

**Table 3:** Survival analysis in denosumab users versus non-users.

		Events [n (%)]		Hazard Ratio (95%CI)	P Value
All-cause mortality	Non-denosumab	78 (48.1)		reference	
	Denosumab	23 (44.2)	Unadjusted	0.61 (0.38–0.98)	0.040
			Adjusted*	0.46 (0.26–0.80)	0.006
CVD	Non-denosumab	32 (19.8)		reference	
	Denosumab	8 (15.4)	Unadjusted	0.54 (0.25–1.17)	0.120
			Adjusted**	0.33 (0.14–0.78)	0.011
Infectious disease	Non-denosumab	26 (16.0)		reference	
	Denosumab	8 (15.4)	Unadjusted	0.63 (0.29–1.41)	0.263
			Adjusted**	0.53 (0.21–1.31)	0.169

Abbreviation: CVD, cardiovascular disease.

adjusted*: Age, sex, diabetes mellitus, dialysis vintage, history of bone fractures, albumin levels, intact parathyroid hormone, calcium, phosphate, C-reactive protein, and vitamin D medication added to the unadjusted model

adjusted**: Age, sex, diabetes mellitus and albumin levels added to the unadjusted model
