# Supplementary figures and images for: Effect of denosumab on the incidence of fractures and mortality in patients undergoing hemodialysis: A retrospective cohort study
Source: PLoS One. 2024 Aug 29;19(8):e0309657. doi: 10.1371/journal.pone.0309657 (PMC11361560; doi:10.1371/journal.pone.0309657)

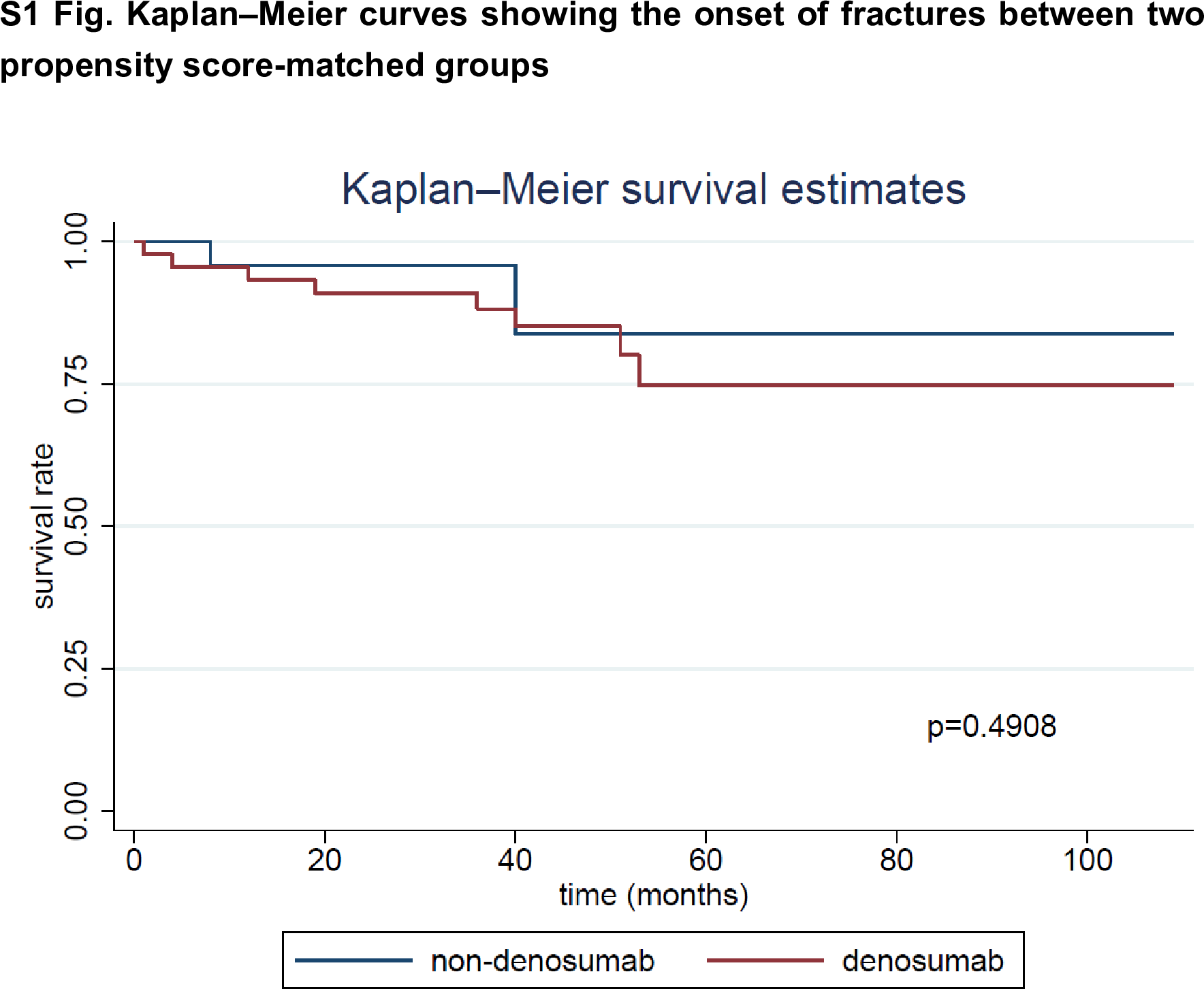

Supplement: S1 Fig — (TIF) [file pone.0309657.s001.tif]

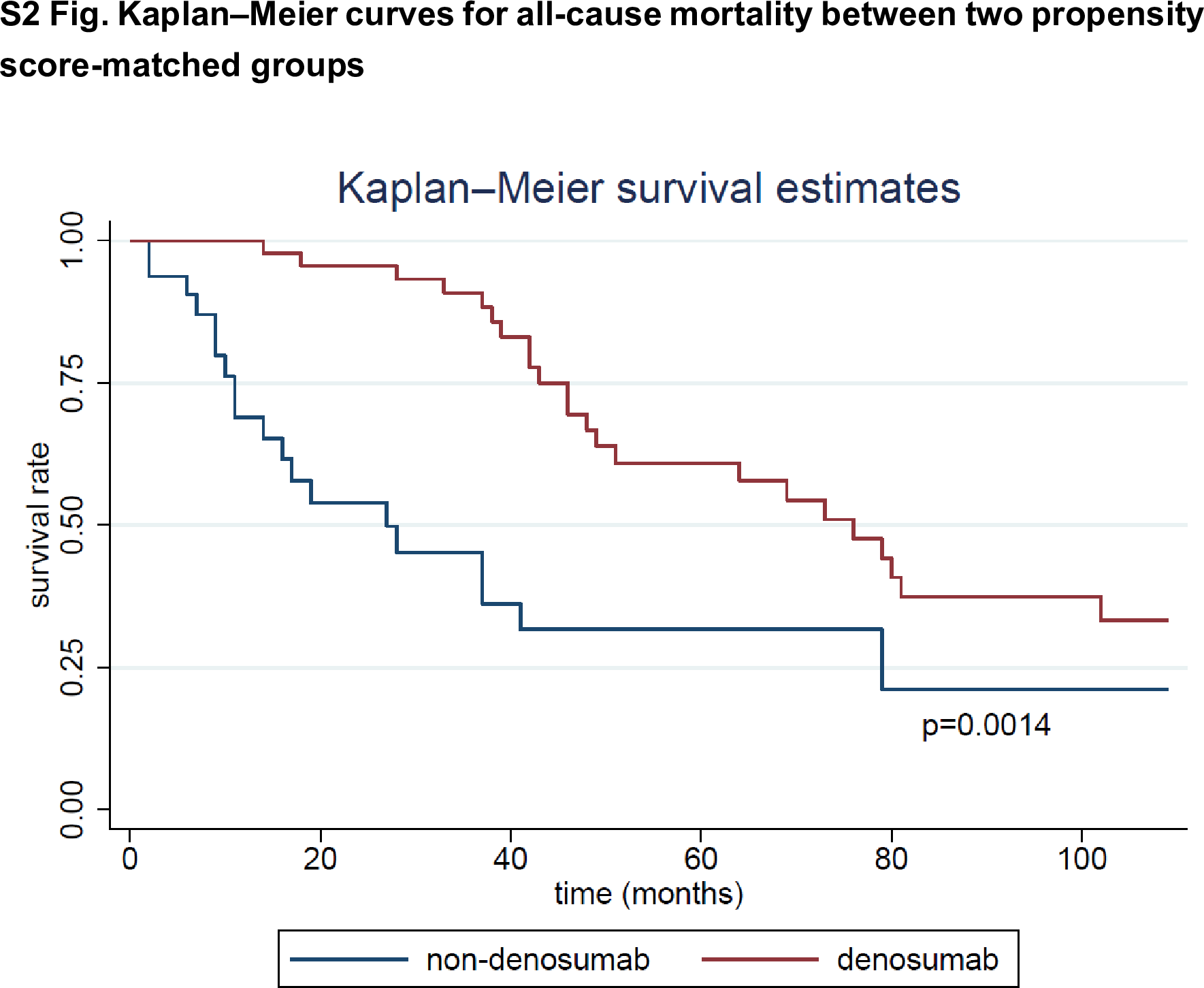

Supplement: S2 Fig — (TIF) [file pone.0309657.s002.tif]

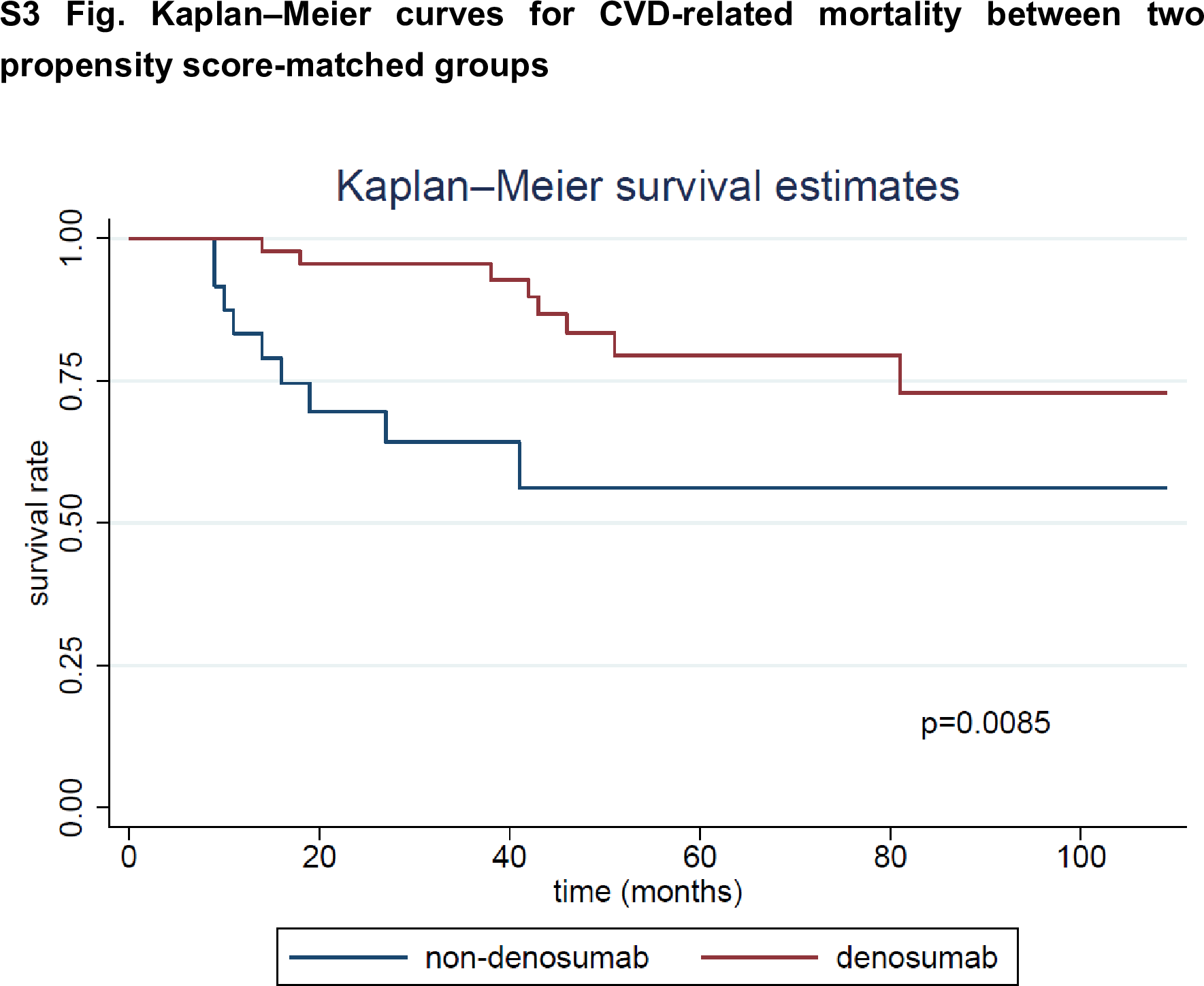

Supplement: S3 Fig — (TIF) [file pone.0309657.s003.tif]

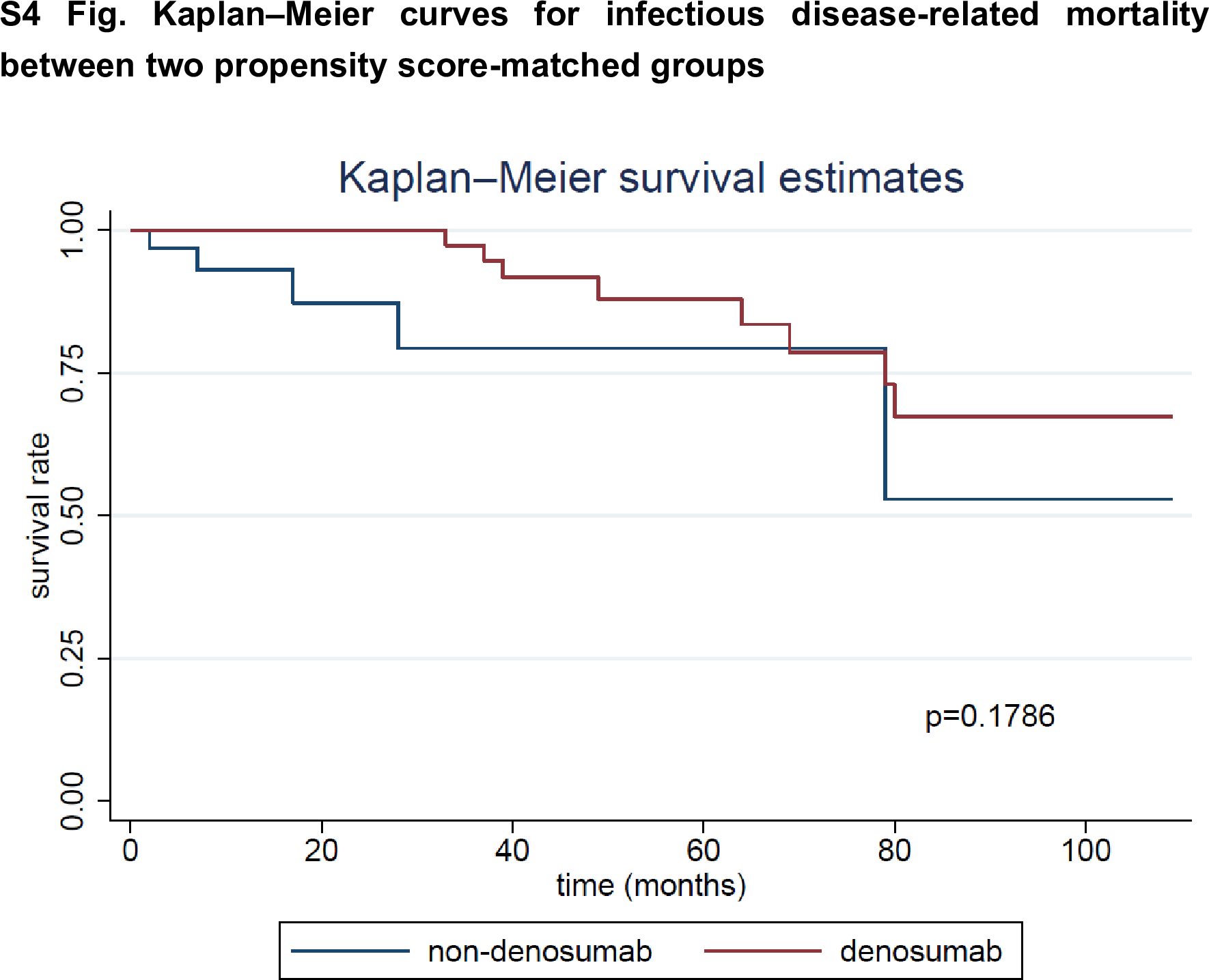

Supplement: S4 Fig — (TIF) [file pone.0309657.s004.tif]
